# Supplementary material for: Increasing HPV Vaccination Uptake among Adolescents: A Systematic Review
Source: Int J Environ Res Public Health. 2020 Oct 30;17(21):7997. doi: 10.3390/ijerph17217997 (PMC7663345; doi:10.3390/ijerph17217997)
Supplement: Supplementary file 1 [file ijerph-17-07997-s001.pdf]

Table S1. Characteristics of the included studies and references.

Author, Year, Country	Study Title	Study design	HPVv Data collection	Study size	Vaccination target age	Strategy target	Description of intervention	Intervention category	Outcome	Proven efficacy (N/Y) Main results
Bundy D.G., 2013 (1) USA	Improving immunization delivery using an electronic health record: the improve project	RCT	Registries	Nr	13–14	HCPs	An electronic alert plus a periodic report on all adolescents late for vaccination for HCPs	Multicomponent	All-doses completion	N
Cassidy B., 2014 (2) USA	A quality improvement initiative to increase HPV vaccine rates using an educational and reminder strategy for parents of preteen girls.	Q-E	Registries	53	11–12	A/P	Informative materials based on the HBM in addition to a telephonic reminder	Multicomponent	First dose; all-doses completion	Y First dose uptake OR = 9.43 (95% CI 2.69–33.10); all doses completion OR = 22.50 (95% CI 4.29–117.99)
Cates J.R., 2011 (3) USA	Evaluating a county-sponsored social marketing campaign to increase mothers' information on HPV vaccine for their preteen daughters in a primarily rural area	C-S	Registries	225	9–8	A/P; HCPs	Social marketing campaign including information on different aspects: product (recommend vaccine against HPV), price (cost, perception of safety and efficacy, and access), promotion (posters, brochures, website, news releases, doctor’s recommendation), and place (doctors’ offices, retail outlets).	Education/Information	Not specified	N

Cates J.R., 2014 (4) USA	Intervention effects from a social marketing campaign to promote HPV vaccination in preteen boys	Q-E	Registries	25,869	11–12	A/P; HCPs	Social marketing campaign including: product (recommend vaccine against HPV), price (cost, perception of safety and efficacy, and access), promotion (posters, brochures, website, news releases, doctor's recommendation), and place (doctors' offices, retail outlets).	Education/Information	First dose	Y Gender specific HR = 1.34 (p = 0.002) for M and HR = 1.09 (p = 0.320) for F
Cates J.R., 2018 (5) USA	Immunization effects of a communication intervention to promote preteen HPV vaccination in primary care practices	Q-E	Registries	147,294	11–13	A/P; HCPs	Focus groups with providers, parents, and preteens, an online training about vaccine epidemiology, communicating with parents and preteens about HPV and HPV vaccine for providers, information materials exposed in clinics for patients	Education/Information	First dose; all-doses completion	Y First dose uptake HR = 1.17 (95% CI 1.1, 1.26); all doses completion HR 1.18 (95% CI 1.07, 1.31)
Choi N., 2017 (6) USA	Successful use of Interventions in combination to improve Human Papillomavirus Vaccination coverage rates among adolescents-Chicago, 2013 to 2015	Q-E	Self-reported	Nr	13–17	A/P; HCPs	Five interventions targeting the public, clinicians, and their staff (1. developing a jurisdiction-wide collaborative initiative with stakeholders; 2. implementing education and skill-building strategies targeting providers; 3. conducting	Multicomponent	First dose; all-doses completion	Y Pre-post data AFIX: first dose uptake increased by a mean of 11% (range, 1%–30%); All doses completion increased by a mean of 7% (range, 1%–30%).

							AFIX enhanced with individualized clinician-to-clinician feedback; 4. implementing IIS-based recall; 5. conducting a comprehensive communication campaign targeting the public)			All interventions: first dose uptake increased by a mean of 20.5% and all doses completion by a mean of 16.1%
Farmar A.L.M., 2016 (7) USA	Achieving high adolescent HPV vaccination coverage	CS	Registries	11,463	13–17	A/P; HCPs	Offering HPV vaccination in bundle with Tdap, MCV4 to all subjects attending a medical visit, internally developed immunization registry20 (VaxTrax) with standing orders/reminder before any visits, in Denver Health	Multicompo nent	First dose; all-doses completi on	Y Gender-specific Compared to national data First dose +32.5% for F and +54.7% for M; All-doses completion +28.4% for F and +38.6% for M (significant 95% CI)
Fujiwara H., 2013 (8) Japan	Community-based interventions to improve HPV vaccination coverage among 13- to 15-year-old females: measures implemented by local governments in Japan	CS	Self-reported	810	13–16	A/P	Several different combinations of the following strategies: school-based strategies, economic incentives, reminders	Multicompo nent	First dose; all-doses completi on	Y First dose uptake ranged from 45.7% for SUB to 74.7% for SUB/DM ($p < 0.001$); from 81.4% for SUB/FT/RC to 95% for SUB/FT/DM/RC ($p < 0.001$); from 95% for SUB/FT/DM/RC to 96.8% for SUB/school based ($p < 0.001$) All doses completion from 3.1% for SUB to 17.6% for SUB/DM ($p < 0.001$); from 73.6%

									for SUB/FT/RC to 90.1% for SUB/FT/DM/RC ($p <$ 0.001); from 90.1% for SUB/FT/DM/RC to 91.2% for SUB/school based (ns) School-based vaccination program with subsidy achieved the highest coverage (96.8% for the 1st dose, 96.2% for the 2nd dose, and 91.2% for the 3rd dose)
Gilkey M.B, 2014 (9) USA	Increasing provision of adolescent vaccines in primary care: a randomized controlled trial	RCT	Registries	107,443	11–18	HCPs	In-person consultation and webinar according to AFIX program	Multicomponent	First dose; all-doses completion	Y/N Age specific Compared to control group First dose for 11-12yo +1.5 pp (+0.3 - +2.7) in person and +1.9 pp (+0.7 - +3.1) webinar; for 13-18yo +0.4 pp (ns) and -0.1 pp (ns) respectively. All doses completion for 11-12yo +0.1 pp (ns) in person and +0.2 pp (ns) webinar; for 13-18yo +0.7 pp (+0.1 - +1.3) and +0.5 pp (ns) respectively

Grandahl M., 2016 (10) Sweden	School-based intervention for the prevention of HPV among adolescents: A cluster randomised controlled study	RCT	Self-reported	741	16–19	A/P	Based on the HBM, school nurses delivered 30 min face-to-face structured information about HPV, including cancer risks and HPV prevention, by propagating condom use and HPV vaccination	Education/Information	First dose; all-doses completion	Y<br>+6.5 pp in the intervention group (52.5 % to 59%)<br>+0 pp in the control group stable at 60.9%
Irving S. A., 2018 (11) USA	Human Papillomavirus vaccine coverage and prevalence of missed opportunities for vaccination in an integrated healthcare system	Q-E	Registries	150	11–17	HCPs	Boosting Recommended Adolescent Vaccination in Oregon' (BRAVO), consisting in 30-minute education session combining information on HPV infection, parental communication strategies, and feedback on clinic- and department-specific coverage data	Multicomponent	First dose; all-doses completion	N
Jacobs-Wingo J. L., 2017 (12) USA	Human Papillomavirus Vaccine uptake: increase for American Indian adolescents, 2013-2015	Q-E	Registries	6239	13–17	A/P; HCPs	Analysis and feedback on vaccine coverage data, educating providers about HPV vaccine, expanding access to HPV vaccine, and establishing or expanding reminder recall and education efforts	Multicomponent	First dose; all-doses completion	Y<br>First dose +24 pp (from 47 % pre to 71% post);<br>All doses completion +22 pp (from 20 % pre to 42 % post)
Kempe A., 2016 (13) USA	Parental choice of recall method for HPV vaccination: a pragmatic trial	RCT	Registries	374	11–17	A/P	Recalling adolescents late for HPV doses. On their choice, during the first-dose appointment, parents who wanted to receive reminders were	Remind	All-doses completion	N<br>Completion rates higher with e-mail and telephone when compared to other

							given a short check-off form clarifying (1) which recall method they preferred (text, e-mail, automated telephone message), (2) if they also wanted a recall sent to their child, and (3) the contact information for their preferred method. The number of recalls parents would receive was not specified			methods (90% vs 60%. *p* = 0.008)
Krantz L., 2018 (14) USA	Increasing HPV vaccination coverage through provider-based interventions	Q-E	Registries	105	13–17	HCPs	Educational seminar and training on communication for HCPs, monthly feedback on vaccination rates to participating clinics, alert for HCPs at any visit for unvaccinated adolescents	Multicomponent	First dose; all-doses completion	Y<br>Gender specific<br>First dose uptake from 83.4% pre to 88.4% post (*p* = 0.02) for M and from 89.8% pre to 91.5% post (*p* = 0.43) for F;<br>All doses completion from 50.9% pre to 61.7% post for all (*p* < 0.05), from 42.6% pre to 57.3% post (*p* < 0.001) for M and from 60.0% pre to 66.5% post, *p* = 0.04) for F
Lee H., 2018 (15) USA	Using narrative intervention for HPV vaccine behavior change among Khmer mothers and daughters: a pilot	RCT	Self-reported	19	14–17	A/P	A storytelling narrative intervention videos which focused on a series of HPV vaccination-related messages based on two theories: rNEM	Education/Information	First dose	N

	RCT to examine feasibility, acceptability, and preliminary effectiveness						and the Storytelling Narrative Communication theory			
Mantzari E., 2015 (16) England	Financial incentives for increasing uptake of HPV vaccinations: a randomized controlled trial	RCT	Registries	1000	16–18	A/P	Financial Incentives and remind intervention	Multicompo nent	First dose; all-doses completi on	Y First dose uptake OR 1.63 (95% CI 1.08–2.47); previous non-attenders OR 2.65 (95%CI 1.61–4.38); All doses completion OR 2.15 (95%CI 1.32–3.50); previous non-attenders OR 4.28 (95% CI 1.92–9.55)
McLean H. Q., 2017 (17) USA	Improving Human Papillomavirus vaccine use in an integrated health system: impact of a provider and staff intervention	Q-E	Registries	16,041	11–17	A/P; HCPs	In-person provider and staff education, quarterly feedback of vaccine coverage, and system-wide changes to patient reminder and recall notifications	Multicompo nent	First dose; all-doses completi on	Age-specific First dose uptake for 11–12yo from pre 40.6% to 59.3% post in intervention group vs 31.9% pre to 44.5% post in the control group (p = .0001). For 13-17yo from 53.0% pre to 61.7% post vs from 48.4% pre to 55.4% post ($p <$ 0.001); All-doses completion for 11–12yo from 32.0% pre to 52.7% post in intervention group vs from 31.6% pre to 52.3% post (ns)

										for the control group; For 13-17 yo from 59.4% pre to 71.9% post and 55.5% pre to 66.9% post, respectively (*p* = 0.08)
Parra-Medina D., 2015 (18) USA	Promotora outreach, education and navigation support for HPV vaccination to Hispanic women with unvaccinated daughters	Q-E	Self-reported	372	11–17	A/P	HPV vaccine educational brochure in individuals' preferred language (English or Spanish) and invitation to participate in the Entre Madre e Hija (EMH) program, a culturally relevant cervical cancer prevention program including 1 hour group health education, referral and navigation support from a promotora (a trained, culturally competent community health worker) and from student peer educators delivered to dyads of mothers and daughters, separately	Education/Information	First dose; all-doses completion	Y<br><br>No difference for first dose uptake.<br>All doses completion increased with AdjOR 2.24 (95% CI 1.25–4.02)
Paskett E.D., 2016 (19)	Results of a multilevel intervention trial to increase human papillomavirus (HPV) vaccine uptake among adolescent girls	RCT	Registries	337	9–17	A/P; HCPs	Clinic-level intervention: Posters, brochures, and tabletop tent cards for the HPVv intervention in waiting room and examination room. Provider-level intervention: 1-hour educational	Multicomponent	First dose	Y<br>Time-specific uptake at 3 months f-up 7.7% intervention group vs 3.2% control group (*p* = 0.061). Uptake at 6 months f-up

							session for providers (current EB HPVv information and strategies for discussing vaccination with parents. Parents-level intervention: An initial phone call, a mail packet including a $10 thank you gift card for completing the telephone-administered baseline survey, an educational brochure and DVD video about HPV and HPVv, a magnet reminder to get the 2nd and 3rd HPV vaccine shots, and a CDC HPVv information statement, a medical record release form and a self-administered survey to return. At the and a $5 gift upon receipt of these items. A second telephone education session about the HPVv			13.1% intervention group vs 6.5% control group (p = 0.002)
Perkins, R. B., 2015 (20)	Effectiveness of a provider-focused intervention to improve HPV vaccination rates in boys and girls	RCT	Registries	7546	11–21	HCPs	Four essential components: 1. repeated contacts with providers, 2. focused education, 3. individualized feedback on vaccination rates, 4. strong quality	Multicompo nent	First dose; all-doses completi on	Y Gender-specific First dose uptake OR 1.6 (p < 0.001) for F and OR 11 (p <0.001) for M;

							improvement incentives in the form of maintenance of board certification requirements			All doses completion OR 1.4 ($p < 0.05$) for F and OR 23 ($p < 0.05$) for M
Pot M., 2017 (21)	Effectiveness of a web-based tailored intervention with virtual assistants promoting the acceptability of HPV vaccination among mothers of invited girls: randomized controlled trial	RCT	Registries	8062	12	A/P	Developing a website providing mothers with tailored feedback (a health communication strategy in which messages are individualized to the person's preferences and needs) from 2 virtual assistants (mother or doctor like assistant)	Education/Information	First dose	N
Rand C. M., 2015 (22)	Effectiveness of centralized text message reminders on human papillomavirus immunization coverage for publicly insured adolescents	RCT	Registries	3812	11–16	A/P	Text message reminders	Remind	First dose	Y<br>HR = 1.3 (95% CI 1.0–1.6)
Rand C.M., 2017 (23)	effects of phone and text message reminders on completion of the Human Papillomavirus vaccine series	RCT	Registries	749	11–17	A/P	Telephonic or text message reminders	Remind	All-doses completion	Y<br>Second dose HR ranged from 1.45 (95% CI 1.04–2.01) to 1.64 (95% CI 1.21–2.23)<br>Third dose HR ranged from 1.72 (95% CI 1.04–2.85) to 2.34 (95% CI 1.67–2.27)

Rand C.M., 2018 (24)	A learning collaborative model to improve Human Papillomavirus vaccination rates in primary care	Q-E	Registries	50	11–17	HCPs	A webinar for providers addressing how to give strong recommendation about HPVv, a report on MO and electronic remind or standing orders	Multicompo nent	First dose	Y<br>Gender specific<br>From 66% pre to 74% post for F ($p < 0.01$), from 57% pre to 65% post for M ($p < 0.01$) and from 62% to 70% for both ($p < 0.01$)
Rehn M., 2016 (25)	Highest vaccine uptake after school-based delivery—A county-level evaluation of the implementation strategies for HPV catch-up vaccination in Sweden	Ecologi c	Registries	325,229	10–12	A/P	Different combinations on strategies compared in different counties including an information channel (App for smartphone, media coverage, county website, school-based information, social media, alert, invitation letter, cinema, YouTube channel) and school-based vaccination	Multicompo nent	Any dose	Y<br>Vaccination in school IRR=1.3 (95% CI 1.1–1.5); school-based information IRR = 1.3 (95% CI 1.1–1.3)
Rickert V.I., 2015 (26)	School-based HPV immunization of young adolescents: effects of two brief health interventions	RCT	Registries	445	≤ 14	A/P	Short messages with rhetorical questions with a technic called "foot-in-the-door"	Education/In formation	First dose; all-doses completi on	Y<br>First dose<br>RR= 1.15 (95%CI 0.89-1.50);<br>No results reported for all-doses completion
Ruffin M. T., 2015 (27)	Impact of an electronic health record (EHR) reminder on Human Papillomavirus (HPV) vaccine initiation and timely completion	CS	Registries	15,021	9–18	HCPs	Systematic electronic reminder for the provider on vaccination at any medical visit	Remind	First dose; all-doses completi on	Y<br>Significant OR according to ethnicity ranging from 2.0 to 3.5 for first dose and from 10.1 to 10.4 for all-doses completion

Sanderson M., 2017 (28)	Pragmatic trial of an intervention to increase human papillomavirus vaccination in safety-net clinics	NRT	Registries	405	9–18	A/P; HCPs	Personalized educational video with possibility to make questions for adolescents. One-hour training session for providers	Education/Information	First dose; all-doses completion;	Y AdjOR was 1.18 (95% CI 0.87–1.60) for the first dose and 0.50 (95% CI 0.29–0.88) for all doses completion
Staras S.A.S., 2015 (29)	Increasing Human Papillomavirus vaccine initiation among publicly insured Florida adolescents	Q-E	Registries	6123	11–17	A/P	Gender-specific postcard campaign and/or in-clinic HIT system	Education/Information	First dose	Y Gender-specific Postcard AdjOR 1.6 (95% CI 1.1–2.4) for F and 1.1 (95% CI 0.8–1.5) for M; HIT AdjOR 1.3 (95% CI 0.9–2.0) for F and 1.4 (95% CI 1.0–2.0) for M; Both interventions AdjOR 2.0 (95% CI 1.1–3.7) for F and 1.6 (95% CI 1.0–2.5) for M
Szilagyi P. G., 2011 (30)	Effectiveness of a citywide patient immunization navigator program on improving adolescent immunizations and preventive care visit rates	RCT	Registries	4115	11–15	A/P	Three steps reminder interventions: Step 1: Patient Tracking Step 2: Reminders/Recall Step 3: Home Visits	Remind	First dose; All-doses completion	Y First dose uptake AdjRR 1.4 (95% CI 1.2–1.5); All doses completion AdjRR1.5 (95% CI 1.4–1.7)
Szilagyi P.G., 2013 (31)	A randomized trial of the effect of centralized reminder/recall on immunizations and preventive care visits for adolescents	RCT	Registries	7546	10.5–17	A/P	Mail or telephonic reminder	Remind	First dose	Y HR ranging from 1.1 to 1.6 ($p < 0.05$)

Tiro J.A., 2015 (32)	Promoting HPV vaccination in safety-net clinics: A randomized trial	RCT	Registries	814	11–18	A/P	1. Information materials specific on HPV via mail 1 - 2 weeks before the first visit. 2. Recall 2 weeks after for non-attenders 3. Recall for the other doses for attenders	Multicomponent	First dose; all-doses completion	Y No effect on first dose uptake; All doses completion rate AdjOR 1.99 (95% CI 1.16–3.45)
Vanderpool R.C., 2015 (33)	Implementation and evaluation of a school-based Human Papillomavirus vaccination program in rural Kentucky	Q-E	Registries	935	9–12	A/P	School-based intervention including Educational materials and informed consent for parents; Telephonic contact with parents to inform about the program and to remind the appointment for vaccination. Information materials for students (articles, website, t-shirt, school events as pizza party etc.) Active student involvement in materials development.	Multicomponent	First dose; all-doses completion	Y First dose uptake increased from 3% pre to 70% post; All doses completion increased from 10% pre to 62% post
Varman M., 2018 (34)	Human Papilloma Virus vaccination among adolescents in a community clinic before and after intervention	Q-E	Registries	3393	9–18	A/P; HCP	Team discussions and staff training for the providers. Reminder mail for parents. Information materials for parents/adolescents. Pictures and information provided for utilization in patient rooms	Multicomponent	All-doses completion	Y Gender-specific All doses completion increased from 50.6% pre to 54.0% post in F and from 39.1% pre to 42.9% post in M

Venturelli F., 2017 (35)	Association between mothers' screening uptake and daughters' HPV vaccination: a quasi-experimental study on the effect of an active invitation campaign	Q-E	Registries	4567	11–12	A/P	Invitation for vaccination with information materials. Two consecutive reminders for non-attenders.	Multicomponent	First dose; all-doses completion;	Y First dose uptake increased from 46.3% pre (95% CI 44.2–48.4) to 77.9% post (95% CI 76.2–79.6). All doses completion increased from 43.9% pre a 74.9% post (No significance reported)
Whelan N. W., 2014 (36)	Engaging parents and schools improves uptake of the human papillomavirus (HPV) vaccine: examining the role of the public health nurse	CS	Registries	3219	Nr	A/P	Adolescents', parents', and schools' engagement by a nurse team specialized in multicomponent school-based vaccination program lead by Public Health Nurses	Multicomponent	First dose; all-doses completion;	Y Sending a remind to parents and teachers' engagement increased first dose uptake with an OR ranging from 1.63 (95% CI 1.09–2.42) to 3.23 (95% CI 7.04–1.48) but not all doses completion (OR 0.56; 95%CI 0.38–0.83). PHN led to increase all doses completion with an OR of 1.65 (95% CI 1.07–2.55)
Winer R. L., 2016 (37)	A cluster-randomized trial to evaluate a mother-daughter dyadic educational intervention for increasing HPV vaccination coverage in American Indian girls	RCT	Registries	97	11–12	A/P	Dinner with power point presentation on HPV and information brochure	Education/Information	First dose; All-doses completion	Y First dose uptake RR = 1.8 (95% CI 0.8–4.4); All doses completion RR = 3.0 (95% CI 0.8–10.8)

Zimmerman R. K., 2017 (38)	Improving adolescent HPV vaccination in a randomized controlled cluster trial using the 4 Pillars (TM) practice Transformation Program	RCT	Registries	10,861	11–17	A/P	Four-Pillar Transformation Program (TP) Practice: Pillar 1 - Convenient vaccination service; Pillar 2 - Communication with parents on importance of the vaccination and its availability; Pillar 3 - Facilitating appointments system; Pillar 4 - Increasing the motivation using Champion one-on-one communication	Multicomponent	First dose; All-doses completion	Y First dose uptake increased from 52.5% to 62.7% (+10.2PP) in the intervention group and from 61.8% to 69.1%, (+7.3PP) in control group. All-doses completion increased respectively of 12.8PP and 12.7PP

Abbreviations: Y Yes; N No; Nr Not reported; Ns Not significant; F females; M males; A/P Adolescents/Parents; HCPs HealthCare Providers; RCT Randomized Controlled Trial; CS Cohort Study; C-S Cross-Sectional Study; Q-E Quasi-Experimental; HPVv HPV vaccination; AFIX Assessment, Feedback, Incentives and eXchange; HBM Health Belief Model; HIT Health Information Technology; MO Missing Opportunities; PP percentage point; IIS Immunization Information System; SUB subsidy, FT free ticket, DM direct mail, RC recall; rNEM revised Network Episode Mode
